# Monthly Summary

**Published:** 1886-07

**Authors:** 


### Monthly Summary.

HOW TO BECOME THIN.—The "beefsteak and hot water cure" for obesity is thus described by Dr. Solomon Smith in the *Lancet*. The course consists in drinking nothing but hot water, and eating nothing but animal food for seventeen weeks. The water is taken in four doses daily, at a temperature of from 130° to 150° Fahr., on an empty stomach, and at least one hour before a meal. The daily average of solid food is 5 lbs., chiefly lean beef; a little plain boiled codfish occasionally.

A patient describes the effect of this treatment as follows: "Two years ago, I weighed (dressed) 16 st. 4 lbs., and my figure was of tubby, aldermanic contour, I am now 13 st. 2 lbs. My waist girth was 44½ inches; now it is 35 inches. I suffered from chronic heart-burn; I have had none of it for fifteen months. I went in daily fear of painful kidney attack; I have not had a symptom of it since I began the hot water. I sleep better, and do both my mental and physical work more easily, and, in fact, feel a much younger man than formerly."

The first thing to bear in mind is that the water is essential; without it, the meat would kill; the next is, that the success of the treatment depends on the possession by the patient of fairly capable kidneys; and the next, that the quantity of meat prescribed is for the cure of the obesity, and not necessarily for that of the lithæmia, the hot water being often useful in cases of lithæmia in conjunction with a mixed diet, the great condition being that no fluid should be drunk at meals.

In the treatment of the lithæmic condition, one finds one's self constantly on the horns of a dilemna, if meat is ordered, the malady is aggravated; if vegetables, the patient suffers from flatulent indigestion, hence the compromise usually recommended; namely, as little meat as the patient can get on with, and such vegetables as he can digest, the whole tempered by occasional blue pills and salines. It must be distinctly borne in mind that this course of diet must not be recommended until we have assured ourselves of the capacity of the patient's kidneys as excretory organs.—*N. W. Lancet.*

---

PROFESSOR HUXLEY ON SMOKING.—At a certain debate on smoking among the members of the British Association, Professor Huxley told the story of his strugglings in a way which utterly put the anti-tobacconists to confusion. "For forty years of my life," said he, "tobacco had been a deadly poison to me. [Loud cheers from the anti-tobacconists.] In my youth, as a medical student, I tried to smoke. In vain! At every fresh attempt my insidious foe stretched me prostrate on the floor. [Repeated cheers.] I entered the navy. Again I tried to smoke, and again met with defeat. I hated tobacco. I could almost lend my support to any institution that had for its object the putting of tobacco-makers to death. [Vociferous cheering.] A few years ago I was in Brittany with some friends; we went to the inn; they began to smoke and looked very happy, and outside it was very wet and dismal. I thought I would try a cigar. [Murmurs.] I did so. [Great expectations.] I smoked that cigar—it was delicious! [Groans.] From that moment I was a changed man, and now I feel that smoking in moderation is a comfortable and laudable practice, and is productive of good. [Dismay and confusion of the anti-tobacconists. Roars of laughter from the smokers.] There is no more harm in a pipe than there is in a cup of tea. You may poison yourself by drinking to much green tea, and kill yourself by eating too many beefsteaks. For my own part, I consider that tobacco, in moderation, is a sweetener and equalizer of the temper. [Total rout of the anti-tobacconists, and complete triumph of the smokers.]—*Medical and Surgical Reporter.*

---

DISTINGUISHED HONOR.—It is, doubtless, a matter of gratification and pleasure to all true members of the dental profession, to know that a high honor has been conferred upon one of its members, one too, for whom the universal suffrage of the profession of this and every other country would have been rendered, had the question been one open for such an expression.

John Tomes has been knighted by Her Majesty, England's sovereign, the second dentist of the world to receive such a distinction. The first was Sir Edwin Sanders, whom all were delighted to have thus honored. In certain lines of scientific and practical work, Sir John Tomes has done more than any man now living. It may be said that he has had great opportunities, but he has improved these as scarcely any one else would have done. It may, with equal truth, be said, that the period of his greatest labor was one in which there were great adverse environments. Embarrassments were met and overcome by him that the dentists of this day know little or nothing about.

The facilities for the prosecution of his work were meager indeed as compared with those of the present. He had not the stimulus and moral support in the recognition and appreciation of dentistry that now obtains.

In spite of difficulties, he achieved a reputation and position in his chosen profession second to none in his own or any other country.

He has done more than any one else to secure for the dental profession a legal recognition and status. He formulated, and had enacted, the best law for the regulation of dental practice that has as yet appeared.

Sir John Tombs has conducted the great work of his life without exciting any special antagonisms, prejudices, or jealousies, which is one of the best evidences of a great and good man.—*Dental Register.*

---

FINAL RESULTS OF OPERATION FOR CANCER OF THE LIP.—Interesting statistics concerning the therapeutic results of the surgery of this affection, are collected from the clinic of Bruns, Tuebingen, by A. Woerner. From an abstract in the *Centralblatt f. Chirurgie*, we learn that of 305 cases operated upon at Tuebingen, the affection occured in females as compared to male patients in the relation of one to nine. The average age was 62 years. In 236 cases no mention is made as to the tobacco smoking habits of the patients. Fifty-one were inveterate pipe-smokers. In eleven cases a trauma was the first origin ; in seven cases neglected warts became the seat of the first start. In sixteen cases the upper lip was affected. Of the 305 cases 217 were operated upon by 354 different operations. Simple excision and union seemed sufficient in 224 cases ; in 28 resection of the maxilla was necessary to total removal. An interesting statement is that one male patient, whose case was inoperable, and who was treated by electrolysis, developed a violent erysipelas. With the subsidence of this, the tumor had almost vanished. The man had no return and died one and a half years after from other causes. Of the 277 operated patients recurrence of the malignant growth ensued in 111 persons; 87.2 per cent. recurred inside of a year, 12 8 after a year. Of the 160 cases that showed no return, 71 or 25.6 per cent. died from other causes, after an average duration of life after the operation of 8.4 years. The 89 that still lived averaged 5.8 years since the operation. Of the whole number, 160 cases, 106 lived over three years ; hence the latter number certainly may be considered as permanently cured. Woerner also compares the previous reports on this subject made by Thiersch v. Bergmann, Billroth. Kocher, etc. The sum of these observations is 866 cases. These patients show results very close to the figures of Woerner. Of the whole number of returns, 87.6 occurred within the first year. The per centage of those that survived three years without a return was 28.1 per cent. as compared with 38.2 per cent. at Brun's clinic.

---

Fun Among the Doctors.—One of the most amusing things in connection with the welcome given the members of the American Medical Association by the citizens generally—was a window piece gotten up by Fraser, the Candy Man, at 609 Olive. He obtained a large number of young ducks, had their skins stuffed and mounted, and seated them in chairs in a little amphi-theatre. The desk in front of each was marked with a parody upon some quack nostrum, and advertisements of patent remedies graced the walls. Among the delegates was a black duckling labelled "hoodoo." The whole was entitled "The other Convension." The hit was palpable and enjoyed by the visiting medicos.—*St. Louis Med. and Surg. Journal.*

